# Materiality in Julio Cortázar’s literature—rereading “Axolotl,” “No se culpe a nadie” and the almanac books

**DOI:** 10.1007/s11059-020-00555-w

**Published:** 2020-09-21

**Authors:** Verónica Abrego

**Affiliations:** Martin-Luther-Universität Halle-Wittenberg, Halle, Germany

**Keywords:** Julio Cortázar, Short stories, Argentina, Critique of anthropocentrism, Otherness, Collage

## Abstract

In the light of the material turn in the Humanities some aspects of Julio Cortázar’s (1914–1984) work become very evident today as a laboratory of the future. For Cortázar, reading was a transforming impulse, part of a process of liberation from mental ties to which he contributed as an author, challenging the barrier between the fantastic and the real, the limit between the human and the animal, between the living and the inert. Thus, as a critic on blind Modernity, Cortázar, from his stories, questions anthropocentrism in a gesture that in the current crisis of the Anthropocene could not be more topical. Moreover, while he supported the transformation of people’s material conditions of life in Latin America, he innovated and celebrated literature in intermedial texts that are the decanted result of a creative performance, embedded in the strongly transcultural context of a diaspora that was first voluntary and then became exile.

What clues does Julio Cortázar’s literary work^1^ offer to focus on materiality? And vice versa, is it possible to discover new aspects in Cortázar’s oeuvre in light of Humanities’ current debates on materiality and the new material turn? Three levels of access to these issues are distinguished here. The first is comprised of his short stories, the best-known texts of his literary work. They build a passage from everyday life to extraordinary existence, attributing to objects and animals transformative qualities that challenge evidence. Stories like “Axolotl” (1956)^2^ and “No se culpe a nadie” (1964),^3^ among others, question anthropocentrism through the awakening of Things and the transmutation of the Living.

*La vuelta al día en ochenta mundos* (1967) and *Último round* (1969), two of his almanac books, are considered here as a second level of access. In them, Julio Cortázar transgresses standard editorial models and literary genres, inviting his readers to take part in the creative process that begins by simply touching a few of the infinite triggers that reality constantly offers.

Finally, by taking into account its biographical anchorage, Cortázar’s work presents a third level of access in terms of its materiality. The author developed his literature as part of a personal search that emerged from the metaphysical-aesthetic world and eventually became part of a historical process that aimed to change the material conditions of reality (cf. i.e. “Acerca de Rayuela,” Cortázar in Bernárdez and Álvarez Garriga 2009, pp. 173–174). Thus, the following pages focus on these three perspectives in order to unravel how Cortázar “mentors” the reader into freedom through the materiality of his texts.

## Materiality and its traces in Julio Cortázar’s literature

Julio Cortázar was born in Brussels (1914), raised and educated in Argentina, and passed away in Paris (1984). As an author, he consciously proposes to the public an epistemic and aesthetic gain through his literature, subverting the logic by which we usually perceive and construct reality. This is one of his most crucial objectives and can be traced back to his initial aesthetic searches; later, it even became one of his goals for political transformation. The study of his manuscripts might give an idea about how the author leads the reader into a different perception of reality, since all writing activity encompasses ordering procedures that allow for the materialization of ideas (cf. Raible 2004, p. 198). As Sybille Krämer and Wolfgang Raible state, authors become their own readers along a way in which the “unordered and simultaneous whole of our mental ideas and thoughts” (Krämer 2004, p. 27) is concretized, put in order and externalized into a medium; then reconstructed, checked and compared with the intention underlying the process (cf. Raible 2004, p. 202). This process is not limited to literary authorship; to a certain degree, it can also be extended to any usual writing activity (cf. id., pp. 200). Based on the analysis of works by Marcel Proust, Émile Zola and Honoré de Balzac, Raible unravels the way in which the ideas of writing experts become text and how an epistemic—and aesthetic—gain is produced in this process (cf. ibd., pp. 202–212).

Unfortunately, among the extensive bibliography on Julio Cortázar, there is not yet any thorough study dealing with the genesis of his work on the basis of his manuscripts, since they are located in Harvard, Princeton and Austin and access to them is restricted.^4^ Nevertheless, and despite its refinement, according to his friend and critic Saúl Yurkievich, Cortázar’s prose, and especially his short stories, seem to have been born from an impulse and written “in one go, as matter and form are generated together” (Yurkievich [1994] 2004, p. 250). Yurkievich specifies: “I’ve had the chance to see his manuscripts and verify that his stories were born from a trance that shaped them completely. There are almost never second or third versions and corrections are always scarce.” (ibid.). Cortázar had claimed to reflect little or nothing when writing a story or poem, to have the impression that “they had written themselves” (id., p. 211); unlike his novels, whose writing process have been more systematic “to carry out action delayed by reflection” (Cortázar [1967] 1995, p. 41). In “Del cuento breve y sus alrededores,” a brief essay on literary work and the ‘handicraft’ of short stories found in *Último round* (Cortázar [1969] 2009a, pp. 59–82), his reflections focus mainly on the closeness of the characters and the feeling of anguish and liberation that writing produces. According to the author, stories are creatures that breathe (id. 78), from which—and not through them—the communication between author and reader takes place, being the author, in this process, the first reader in amazement of himself (cf. id., p. 79). Through his unorthodox way of ordering reality, Cortázar builds a close relationship with the reader, from whom he needs complicity and shrewdness in order to invoke the revealing power of imagination (cf. Yurkievich [1994] 2004, p. 250).

The recent material turn occurring in the Humanities allows us to appreciate a further fundamental aspect of Cortázar’s work, one which exposes his contemporaneity and postulates once again the role of literature as a laboratory of the future. The so-called new materialisms focus on matter and on the objectiveness of life as being actively involved in the (re-)production of the world. Relevant features of the new materialisms movement are life processes, which had been detached by the rationalism of Modernity, as well as relations between the human and the non-human; between organic and inorganic forms.

It is precisely its interest in the inorganic that represents the new element of this orientation (cf. Hennessy 2017, p. 99; Coole and Frost 2010, p. 7). Together with cultural post-humanism, i.e. by Rosi Braidotti or Karen Barad, they promote a focus on relations between and throughout the species, as well as on “the vitality and agency of nature” (Coole & Frost, ibid). To these interweaving perspectives should be added the closely linked postulates of Latin America’s “Buen Vivir” and the cosmologies of the native peoples, in which air, water, soil, animals and plants are considered to be invested with agency. This present confrontation of an anthropocentric standpoint is obviously chained to the long process of illuminating the dark side of the subject of Enlightenment (the white European man) and questioning the limits of Cartesian reasoning and its consequent divisions and hierarchies, as well as the construction of an “Other” as a constituent element of Modernity.

This line of questioning, and the social criticism implied by it, is also recognizable in artistic and cultural movements of the early twentieth century, such as Surrealism, which offers an important critique of rationalism in Cortázar’s thought.^5^ The positions held by Surrealists were far from purely aesthetic; rather, their proposals for individual and socio-political transformation went hand in hand with artistic and literary innovations (cf. Vanbroeckhoven 2019, pp. 38–40). As Joel Vanbroeckhoven points out, Cortázar identified with the worldview of the Surrealists, although not with their methods (id., p. 58). Furthermore, from the 1930s onwards, when Surrealism was already “domesticated,” the *Sur* magazine brought Existentialist philosophy to Buenos Aires, especially of French origin, and Cortázar contributed to its dissemination (cf. Yurkievich in Yurkievich et al. [1994] 2017, p. 19). In the intersection between both currents that pervaded in this period, Cortázar formulated his *Teoría del Túnel* (1947, Cortázar in Yurkievich et al. [1994] 2017, pp. 15–112). In it, he proposes that literature is the total expression of the human being, a “modo verbal del ser del hombre” (a verbal mode of man’s being, ibid., p. 57), and that the [true] writer-poet distrusts and challenges language as a “perfecta, infalible máquina de enunciación” (perfect, infallible enunciation machine, id., p. 62). Although the author himself described—retrospectively—this period of his intellectual work as reduced to aesthetic interests, there are several clues that allow us to recognize, from very early on, Cortázar’s wish to promote an integral transformation of the reader through literature—initially, perhaps, relying mainly on aesthetic means. The literary presence of Cortazar’s work bears the stamp of the (*anti*-)novel that consecrated him in 1963, *Rayuela* (engl. *Hopscotch*). In it, Cortázar questions the usual construction of reality at the level of the structure, its literary motifs and the language used. The concerns that he expresses in his masterpiece, however, date from much earlier. After reading his correspondence, it can be argued that his intense friendship with the poet and globetrotter Fredi Guthmann had a profound and lasting impact on the writer and builds a major contribution to his intellectual pursuits. The letter dated from July 26th 1951 reveals Cortázar’s goal set in one of the deep-rooted conventions of Western thought:

A veces, con lo que pueda yo tener de poeta, entreveo fulgurantemente una instancia de esa Realidad: es como un grito, un relámpago de luz cegadora, una pureza que duele. Pero instantáneamente se cierra el sistema de las compuertas; mis bien educados sentidos se reajustan a la dimensión del lunes o del jueves, mi bien entrenada inteligencia se ovilla como un gato en su cama cartesiana o kantiana. Y el noúmeno vuelve a ser una palabra, una bonita palabra para decirla entre dos pitadas al cigarrillo.

No importa, Fredi; mucho es ya saber que esa realidad está ahí, del otro lado.

(Sometimes, with what I may have of a poet, I glimpse an instance of that Reality: it is like a scream, a blinding flash of light, a purity that hurts. But instantaneously the system of floodgates is closed; my well-educated senses are readjusted to the dimension of Monday or Thursday, my well-trained intelligence is curled up like a cat in its Cartesian or Kantian bed. And the noumenon becomes a word again, a nice word to say between two puffs of the cigarette. It doesn’t matter, Fredi; it means a lot to me to know that this reality is there, on the other side. Cortázar in Bernárdez & Álvarez Garriga 2012a, p. 321)

In a letter dated a few months before *Rayuela*’s publication, Cortázar reveals to Fredi Guthmann that the novel is inspired by the dialogues he had with him and that its central character, Horacio Oliveira, reflects “tu humor, tu enorme sensibilidad poética, y sobre todo tu sed metafísica” (your humor, your enormous poetic sensitivity, and above all your metaphysical thirst, Letter from June the 6th 1962, Cortázar in Bernárdez and Álvarez Garriga 2012b, p. 285). Cortázar confesses that, although the novel is only partially autobiographical, “he puesto todo lo que siento frente a este fracaso total que es el hombre de Occidente” (I have put [there] everything I feel in front of this total failure that is the occidental man, ibid.). In fact, after publishing his magnum opus, it is not the “noumenon,” but the socio-historical conditions of Latin Americans that started concerning Julio Cortázar more and more. However, his early reflections on occidental logic and the value of perception and intuition marked his thinking largely to the end, albeit his ideas on transformation having increasingly included, later on, the socio-political and historical spheres, the material conditions of human life. In the prologue to his last long interview, given in 1983, Omar Prego Gadea quotes that Cortázar’s “intention was to hold together what he called ‘the revolution from the outside and the revolution from within’” (1997, p. 32).

## Materiality of an oeuvre in transformation: from the aesthetic liberation to the social-emancipatory premise

In his masterclass on literature, given at Berkeley in 1980, Julio Cortázar looked back on three phases of his development as a writer. He defined them respectively as his aesthetic, metaphysical and historical stages. At the predominantly aesthetic stage, his passion for literature led him to read “the best books” available in Buenos Aires and to write with certain models in sight or with “un ideal de perfección estilística profundamente refinada” (ideals of profoundly refined stylistic perfection, Cortázar in Bernárdez and Álvarez Garriga [1984] 2016, p. 17). At that time, when he was a distant witness of the Spanish Civil War and, later, of the Second World War, Cortázar took sides with the Republicans against Nazism, but he never thought that his role as a writer would allow him to go beyond a position, a commentary or simply beyond his sympathy towards one of the combatant groups (cf. ibid.). Once in Europe, that is, from 1951 onwards, a stage of “autoindagación lenta, difícil y muy primaria” (slow, difficult and very primary self-inquiry, id., p. 20) began. His investigation on the human being “como destino, como camino dentro de un itinerario misterioso” (as a destination, as a path within a mysterious itinerary, ibid.) becomes gradually manifest. This search is recorded in his novels *Los Premios* and *Rayuela*, and is especially embodied in the latter through the aforementioned figure of the protagonist Horacio Oliveira. The third stage starts with his visit to Cuba in January and February 1963, when he was invited to participate in the jury of Casa de las Americas. This visit became a turning point and made Cortázar a fervent defender of the revolutionary cause in Latin America. Years later, in one of his last interviews, Cortázar admitted that until the moment he became politically aware, he had been indifferent to the political ups and downs of the world and its protagonists, “[…] yo me quedaba afuera de la parte que correspondía a la sangre, a la carne, a la vida, al destino personal de cada uno de los participantes en esos enormes dramas históricos” (I had remained outside the part that corresponded to the blood, the flesh, the life, the personal destiny of each of the participants in those enormous historical dramas, Cortázar in Prego Gadea 1997, p. 211). After Cuba and back in his country of residence, France, Cortázar recognized himself not only as an Argentine author, but also as a Latin American. Moreover:

Me di cuenta de que ser un escritor latinoamericano significaba fundamentalmente que había que ser un latinoamericano escritor: había que invertir los términos y la condición de latinoamericano, con todo lo que comportaba de responsabilidad y deber, había que ponerla también en el trabajo literario.

(I realized that being a Latin American writer meant fundamentally that one had to be a Latin American who writes: one had to invert the terms and the condition of the Latin American, with all that this entailed of responsibility and duty, one had to put it also in one’s literary work. Cortázar in Bernárdez & Garriga [1984] 2016, p. 24)

During a time of strong political effervescence and dichotomous positions, imposed by the Cold War, his claim to being a Latin American writer while having chosen to live in Paris (he even applied for French citizenship) earned him profound criticism from the Latin American intelligentsia. Two people were especially harsh moments in their criticism of Cortázar: David Viñas and Liliana Heker. The writer and literary critic David Viñas found Cortázar’s understanding of his place of enunciation as a revolutionary and a Latin American unreliable (cf. Viñas 1971, pp. 27–34; Letter to Saúl Sosnowski, dated September 29, 1972, Cortázar in Bernárdez and Álvarez Garriga 2012d, pp. 318–322; cf. Goloboff 1998, pp. 144–147). Some years later, Liliana Heker would criticize the comfort of his exile in Paris during Argentina’s last dictatorship (cf. Heker 1980, pp. 3–5; Letter to Liliana Heker, dated November 26, 1980, Cortázar in Bernárdez and Álvarez Garriga 2012e, pp. 312–316; Cortázar in Yurkievich 1984a, pp. 16–25).

The central question arisen by these controversies was whether it is possible to consider oneself a Latin American writer living in Europe, under conditions of double loyalty and schizophrenic language (cf. Goloboff 1998, pp. 144–147). Before the mass exiles due to the multiple Latin American dictatorships (through which the condition of exile affected many politically engaged persons), the discussion around the non-resident countryman was based on mistrust and revealed a difficulty in understanding the migrant’s place of enunciation. Moreover, the cultural influence of the European metropolises constituted a common place for cultural criticism and accompanied the discourse of social emancipation. So, for instance, despite having proven themselves to be profoundly anti-fascist during the Second World War, the members of *Revista Sur* were also criticized for their so-called ‘foreign tastes’ and were mainly considered to be part of the cultural establishment, being disliked because of their elitism and conservative or anti-Peronist positions.

For living in Paris and celebrating and vindicating European culture, Cortázar’s resistance and his incessant struggle for the disclosure of crimes perpetrated by the state in Latin America was often ignored by those that considered his situation as privileged when compared to those living under dictatorial societies. Today, Julio Cortázar’s position can be better understood in light of the concept of cultural citizenship and in recognition of the complex processes of cultural translation. His writing on the materiality of existence, the necessary incorporation of the social location, the speaking out from “the blood, the flesh, the life” (see above), is also known in feminist studies as “situated knowledge” (Haraway 1988). In dealing with knowledge positions, Haraway is concerned—above all—with the critical review, deconstruction and interpretation of the dominant knowledge (cf. Haraway 1988, p. 584). In recent decades, these postulates have coincided with the proposal made by Latin American cultural studies to decolonize epistemology, culture and thinking. Cortázar, however, had fought a similar battle much earlier in the literary field, aiming at an integral transformation of the readers:

Creo que a nosotros los escritores, si algo nos está dado—dentro de lo poco que nos está dado— es colaborar en lo que podemos llamar la revolución de adentro hacia afuera; es decir, dándole al lector el máximo de posibilidades de multiplicar su información, no sólo la información intelectual sino también la psíquica, su contacto con los elementos que lo rodean y que muchas veces se le escapan por mala información y por carencias de todo tipo. Si algo puede hacer un escritor a través de su compromiso ideológico o político es llevar a sus lectores a una literatura que valga como literatura y que al mismo tiempo contenga, cuando es el momento o cuando el escritor así lo decide, un mensaje que no sea exclusivamente literario.

(I believe that for us writers, if something is given to us—within the little that is given to us—it is to collaborate in what we can call the revolution from the inside out; that is, giving the reader the maximum possibilities of multiplying his/her information, not only intellectual information but also psychic information, her/his contact with the elements that surround her/him and that many times escape her/him due to bad information and deficiencies of all kinds. If there is one thing a writer can do through her/his ideological or political commitment, it is to bring to her/his readers a literature that is worthwhile as literature and at the same time contains, when the time comes or when the writer decides, a message that is not exclusively literary., Cortázar in Bernárdez & Álvarez Garriga [1984] 2016, p. 34).

His 1980 retrospective look refers to his refusal to write a programmatic literature; or even more, to his fundamental opposition to using literature as a tool for revolutionary causes, or to circumscribe it to social commitment (cf. Letter to Fernández Retamar dated May 10, 1967, Cortázar in Bernárdez and Álvarez Garriga 2012c, pp. 412–421). On the other hand, from the moment he began supporting the Latin American revolutionary causes and, later on, the fight against the region’s dictatorships, his socio-political concerns became more and more clearly manifested in a literature “that aims high” but that, ironically, would earn him the ‘elitist’ label (cf. Goloboff 1998, pp. 231–232).

Beyond all the virtues literature may possess and unleash, Cortázar had no illusions about the influence of literature on reality; however, he did not believe that it was useless (cf. id., p. 227). From the 1970s onwards, when repression became massive in Latin America, Cortázar showed his solidarity through cultural and political assignments. Years of intense social commitment follow, including his participation in the Second Russell Tribunal on Latin America, and from 1979 onwards, supporting the Sandinista revolution against Somoza in Nicaragua.^6^ His political commitment had since been expressed in a series of texts. At first, in 1973, he contributes to the collective work *Chili: dossier noir*, which denounced the violation of human rights by the Chilean military junta. In 1974, he received the Médicis étranger prize for *Libro de Manuel* as the best foreign book published that year, and donated the amount of the prize to the Chilean resistance. Among many other things, the book denounced the escalation of violence under the government of General Lanusse in Argentina.

In 1975 *Fantomas contra los vampiros multinacionales* is published, a very popular comic book edition about the crimes occurring in Latin America at the time. The booklet, the revenue of which was intended to promote the work of the Russell Tribunal, included also its sentences and a summary of its work and ideals. It became available not only in bookstores, but also in newsstands, reaching, in Mexico, a distribution of 60,000 copies within 2 months (cf. Filosofía en español 2020). Two other books that interweave the political with the literary appeared posthumously: *Nicaragua tan violentamente dulce* (1984), the copyright of which Cortázar bequeathed to “the Sandinista people of Nicaragua” (id., p. 6); and *Argentina: años de alambradas culturales* (1984), edited by Saúl Yurkievich, which is a selection made by Julio Cortázar of the articles he had published since 1973 that remained “on the other side of the sieve” (id., p. 7), that is, articles subject to censorship that never reached the Argentine public.

## Untamed materiality in Cortázar’s short stories

Almost all the short stories I have written belong to the genre called “fantastic” for lack of a better name, and they oppose that false realism that consists of believing that all things can be described and explained according to the philosophical and scientific optimism of the eighteenth century.

(Julio Cortázar, “Algunos aspectos del cuento” in Yurkievich et al. [1994] 2017, p. 476,

here in Picon Garfield 1975, p. 12)

As one of the masters of the Latin American short story, Julio Cortázar has referred in different contexts to his positions on the subject. There are, of course, the well-known brief essays “Algunos aspectos del cuento” –written between 1962 and 1963 and published in the Casa de las Americas Magazine in July 1970—(s. Cortázar in Yurkievich et al. [1994] 2017, pp. 475–493) and “Del cuento breve y sus alrededores”—sole theoretical text present in *Último round* (Part I, cf. Cortázar [1969a, b] 2009, pp. 59–82). In the aforementioned last interview with Omar Prego Gadea, Cortázar’s reflections revolved around the sphericity of the short story, its language and musicality. Sphericity is a recurring metaphor for the ultimate form of a short story (cf. Cortázar in Prego Gadea 1997, p. 98): “constituye una esfera cerrada donde se desarrolla una acción dramática” (a closed sphere where a dramatic action takes place, Cortázar in Yurkievich 1984a, pp. 119–120). It’s opposed to the open configuration of his novels or almanac books (cf. Yurkievich 2004, p. 19).

According to his statements, he adopts the Borgesian^7^ lesson of eliminating everything that can be eliminated, that is to say, to write in a tight but not hard way, with economy of words (cf. Cortázar in Prego Gadea 1997, p. 98). “Para mí la escritura es una operación musical” (writing is for me a musical operation, ibid.) is the idea he applies to his short stories, keeping in mind the notion of rhythm, of euphony: “la eufonía que sale de un dibujo sintáctico […] que al haber eliminado todo lo innecesario, todo lo supérfluo, muestra la pura melodía” (the euphony that comes out of a syntactic drawing that, having eliminated all the unnecessary, all the superfluous, shows pure melody, ibid.). Cortázar tries to achieve a so-called purity, here understood as stringency of meaning, for the opening of “[…] la comunicación de lo intuitivo que yo le quiero dar al lector […]” (the communication of the intuitive that I want to give to the reader, id., p. 99). In this sense, the fantastic floods the everyday and appears in his literature as a space of transition to another dimension.

He refers to the fantastic as a nostalgic value that reminds us of a special moment when we are both ourselves and something unexpected; “el momento en que la puerta antes y después da al zaguán se entorna lentamente para dejarnos ver el prado donde relincha el unicorno” (the moment in which the door that before and after leads to the hall slowly turns around to let us see the meadow where the unicorn neighs, Cortázar [1969a] 2009, p. 79). The space of transition emerges when “osmosis,” or “convincing articulation,” of the fantastic occurs (ib., p. 82). For the writer, this is like a balancing act between two poles. On the one hand, in order to be *extra*-*ordinary*, the fantastic has to draw attention inside a common context, because “solo la alteración momentánea dentro de la regularidad delata lo fantástico” (only the temporary alteration within the regularity reveals the fantastic, ib., p. 79). On the other hand, to create the *possible reality effect*, the fantastic must be established for a certain time as a new partial order (cf. ib., p. 81). However, the proportion of this order is not immovable. In his definition of the fantastic, Tzvetan Todorov writes that the fantastic lies in the moment of hesitation, “the text must oblige the reader […] to hesitate between a natural and a supernatural explanation of the events described” (Todorov [1970] 1975, p. 33). He refers to this interstitial instant as the moment in which the reader is not able to discern whether s/he should decide to consider what s/he has read as uncanny (and supernatural) or wonderful (and extraordinary). In an interview with Mario Vargas Llosa, in 1965, Cortázar stated that, as an author, he doesn’t even make such a distinction: “Yo no sé dónde empieza o termina lo real y lo fantástico; en mis primeros libros preferí insertar lo fantástico en un contexto minuciosamente realista, mientras que ahora tiendo a manifestar una realidad ordinaria dentro de circunstancias con frecuencia fantásticas.” (I don’t know where the real and the fantastic begin or end; in my early books I preferred to insert the fantastic into a thoroughly realistic context, whereas now I tend to manifest ordinary reality within often fantastic circumstances. Cortázar in Alazraki 1983, p. 117).

According to Cortázar specialist Jaime Alazraki, the fantastic content of the short stories reveals the same question formulated in his novel *Rayuela*: the confrontation between a secondary reality and an everyday reality that masks it (cf. Alazkari 1983, pp. 211–212). After reviewing the studies on the fantastic done by Roger Caillois and Tzvetan Todorov, and on contemporary art by Umberto Eco, Alazraki showcased that Cortázar exposes the permeability of the border between the real and the fantastic and teaches how “the real” depends extremely on our capacity to perceive the world. With his appreciation of what he calls the *neo*-*fantastic*, Alazraki proposes that Cortázar works with two geometries (1983, pp. 114–120): he starts from an order that we can call the real or the causal, and transcends it, creating “a new order that contains the previous one, but that is also situated above it. From this new perspective, the horizon is widened by discovering planes and relationships invisible from a realistic perspective” (Alazraki 1983, p. 119). Unlike the fantastic, the neo-fantastic does not confront the reader with her/his fears, but with the logic s/he applies to measure the limit between reality and unreality (cf. Alazraki 1983, p. 35). The matter can be summarized in the very own words of the writer, who, on having recognized writers such as Edgar Allan Poe as references to his work, explained: “[…] esa disposición a lo fantástico que me asalta en los momentos más inesperados y que me lanza a escribir como la única manera de cruzar ciertos límites, de instalarme *en el territorio de lo otro*” (this disposition to the fantastic assaults me in the most unexpected moments and hurls me to write as the only way of crossing certain limits, of settling down in the territory of the other, Cortázar in Alazraki 1994, p. 61, my italics).

The following analysis explores precisely these territories of the other. As a relational context of the fantastic, the everyday, the habitual and the conceivable are subject to transformations that, several decades after Cortázar’s creations, allow us now to establish connections with new contexts and meanings in his work. The questioning of anthropocentrism brings together the texts analyzed herein, since: “en el siglo XX nada puede curarnos mejor del *antropocentrismo autor de todos nuestros males* que asomarse a la física de lo infinitamente grande (o pequeño)”^8^ (in the twentieth century, nothing can better cure us from *anthropocentrism, which is the author of all our evils*, than to look into the physics of the infinitely large (or small), Cortázar 1967, p. 25, my italics). Dismissing anthropocentrism and thinking in planetary terms occupies a prominent place in the agendas of the beginning of the twenty-first century. In the age of the Anthropocene, that is to say, in which human beings act as a geological force that largely models and destroys earth, climate, environment and life, it appears adequate to examine Cortázar’s critical imagination against anthropocentrism from the perspective of a new materialism. The Anthropocene concept makes visible the interweaving of nature, society, culture, material life and technology, drawing attention to a human being that, in spite of her/himself, is part of a whole and not its center, nor its culmination point. Both short stories referred to henceforth were published in *Final del juego* (End of the Game), Cortázar’s second collection (cf. Cortázar [1956] 2016). The book was initially published in Mexico in 1956 and included nine stories, among them “Axolotl” (cf. Goloboff 1998, p. 107). *Final del juego’*s current version dates from 1964 and contains material written between 1945 and 1962; most of the stories have in common the persistent theme of the ‘otherness’ (cf. Picón Garfield 1975, p. 30).

- Harbingers of the post-human in “Axolotl” (1956)

There is a huge body of secondary literature based on Cortázar’s short stories. A relatively recent publication includes readings of “Axolotl” that analyze the construction of otherness in terms of animal logic (Gremels 2016) and in relation to the cultural coordinates of the primitive/exotic (Bernales Albites 2016). Other readings of this story have emphasized its double plot (Di Gerónimo 2001), the manipulation of the reader’s gaze (García Moreno 2001) and its character as an exponent of a dialectic between Dantesque and Aztec mythology (Graf 2002)—or as an ethnographic allegory (Camayd-Freixas and González 2000), an idea that had already inspired Knight and Krull to discover a hidden Indian in the story (1973). Furthermore, Planells put his magnifying glass on its communicative configuration (Planells 1976) and Jaime Alazraki analyzed the axolotl mainly as a metaphor (1994, pp. 69–70).

The short stories by Cortázar that establish a relationship between animals and humans have often been read as exhibits of the unreal due to their fantastic effect. But this fantastic effect can also be thought of as an example of the expansion of the limits of perception, as a challenge to the usual cultural coordinates and as ways of conjugating a different relationship with the environment. For Rosi Braidotti, Humanism postulates “otherness as non-human, non-white, non-male, non-young, non-normal, non-healthy, non-heterosexual: it is a minus, a minus-one type of thinking” (Braidotti 2018, 00:05:30). From the perspective of a post-anthropocentric post-humanism, Humanism’s subject is criticizable for constituting himself dialectically through his “other” and instrumentalizing difference to create hierarchies, thus placing himself at the top of that order. The animals represent his counterpart; they occupy the other side, the negative place par excellence: “The animal is the necessary, familiar and much cherished other of *anthropos*.” (Braidotti 2013, p. 68).

On the way to a post-anthropocentric subject, post-anthropocentric post-humanism does not propose to humanize animals, appealing to the moral and legal principle of equality (cf. Braidotti 2013, p. 84), but to recognize that the forms of relationship and interaction existing between animals and humans are constitutive for both and need to be re-explored and transformed:

It is a transformative or symbiotic relation that hybridizes and alters the ‘nature’ of each one and foregrounds the middle grounds of their interaction. This is the ‘milieu’ of the human/non-human continuum and it needs to be explored as an open experiment, not as a forgone moral conclusion about allegedly universal values or qualities. The middle ground of that particular interaction has to remain normatively neutral, in order to allow for new parameters to emerge for the becoming-animal of *anthropos*, a subject that has been encased for much too long in the mould of species supremacy. Intensive spaces of becoming have to be opened and, more importantly, to be kept open. (Braidotti 2013, pp. 79–80).

Other approaches made by post-humanism regarding animals as subjects place on the agenda a human being willing to review her/his relationships with other beings and to take responsibility for the enormous interconnection between *Zoe*, the Living. Thus, among other things, a *Zoe* egalitarianism proposes to rethink our animal companions not only affectively, but organically, as “nature-cultural compounds” (cf. id., p. 73). The story contemplated here also reveals these dimensions.

“Axolotl” is the tale of a man who is fascinated by a species of amphibians of Mexican origin, the axolotls, whom he visits and observes recurrently in an aquarium at Paris’ Jardin des Plantes^9^ until he becomes one of them. In its structure and plot, the story is an example of sphericity as circularity. From the very first paragraph, we become aware of a process that has already taken place and that the story will barely detail. It anticipates the long visits of a man, an independent human subjectivity, to an aquarium, his recurrent thoughts about axolotls and makes an explicit mention of the place of enunciation as a result of the process: the narrator, that is, the one who successfully communicates with the reader, is an axolotl. The last paragraph explains the solitude of an apparently independent axolotl subjectivity and refers to the beginning of the story: outside the aquarium, a narrating self is given the task of telling the story of this encounter which, as we know from the beginning, is not written by a man but by an axolotl.

According to the author, there is a set of his stories that seem to have been born out of his nightmares; however, “Axolotl” emerged from an experience of everyday life (cf. Prego Gadea 1997, pp. 94–96). As the letter to Eduardo Jonquières, dated of June the 14th 1952, shows, the story was written probably in the first 2 weeks of that month,^10^ during Cortázar’s first phase of cultural exploration of Paris, a very productive and intensive period (cf. Cortázar in Bernárdez and Álvarez Garriga 2012a, p. 381). The author declares having had a terrifying experience looking at the axolotls. Asked thirty years later about the genesis of the story, Julio Cortázar recalls his fear and the desire to escape from the attraction of the axolotls that afternoon in the Jardin’s aquarium:

[…] “Vos sentís que no hay comunicación, pero al mismo tiempo es como si te estuvieran suplicando algo. Si te miran es que te ven, y si te ven, qué es lo que ven. En fin, toda esa cadena de cosas. Y de golpe tener la impresión de que hay como una ventosa, un embudo que te podría embarcar en el asunto.

Y entonces huir. Yo huí. Y esto es absolutamente cierto; será un poco ridículo pero es completamente cierto: jamás he vuelto al acuario de Jardin des Plantes, jamás me voy a acercar a ese acuario.”

(You feel that there is no communication, but at the same time it is as if they are begging you for something. If they look at you, they see you, and if they see you, what do they see? Anyway, that whole chain of things. And all of a sudden you get the impression that there’s like a suction cup, a funnel that could get you into it. And then run away. I ran away. And this is absolutely true; it may be a bit ridiculous but it is completely true: I have never gone back to the aquarium of the Jardin des Plantes, I am never going to go near that aquarium. Cortázar in Prego Gadea 1997, p. 95)

The story “Axolotl” is an experiment in which the writer uses the power of his imagination to unwind what would have happened had he exposed himself more openly to the sensation caused by the amphibians, making permeable the boundary between both living materials, as he had intuited. The story postulates a disturbing thesis: between human beings and axolotls, there is a vital continuity. It is a tailor-made example of *Zoe* egalitarianism, a story that explores the boundaries between species, permeating them in a process that ends in fusion.

How does this transmutation between axolotl and man take place in a story that has only eleven paragraphs? Unlike the Kafkaesque Gregor Samsa, who wakes up one morning transformed into an insect, the narrative self of “Axolotl” describes the process meticulously, although the decisive moment takes place suddenly. The gradual process of erasing the borders between the species has its correlation in two operations: the positioning of the narrator outside and inside the aquarium—in and out of the axolotl’s matter and subjectivity—at the same time that a speaking from the body sediments the coherence of his place of enunciation.

The narrative situation is a complex one. The narrative self also occupies the position of a third-person narrator, who is both witness/observer and omniscient narrator; that is, he places himself both inside and outside the narrated events. Initially, the narrative self relates with a human subjectivity and from a spectator position; but, up to the fourth paragraph, s/he/it suddenly becomes the one explaining from the body of the amphibian, though with a subjectivity that exposes a human behavior by the axolotls. In one sentence, the narrator is outside the aquarium and, in the next, seamlessly inside:

Once in a while a foot would barely move, I saw the diminutive toes poise mildly on the moss. It’s that we don’t enjoy moving a lot, and the tank is so cramped—we barely move in any direction and we’re hitting one of the others with our tail or our head—difficulties arise, fights, tiredness. The time feels like it’s less if we stay quiet. (Cortázar [1956] 1998, p. 162).

The narrator assumes the position of a human spectator again in the fifth paragraph: “some of them swim with a simple undulation of the body” (ibid.). But, after anticipating what will happen (“They were not *animals.*” (ibid.), the spectator-narrator self-compiles historical and mythical causalities to elucidate the transmutation that has been announced by describing a human kind of obsession, a desire to be there where the axolotls are located (“lt got to the point that I was going every day, and at night I thought of them immobile in the darkness,” id., p. 163). After the transmutation, the narrator ceases to be a spectator to become an axolotl’ subjectivity; at first hesitantly, showing a certain ambivalence between the mental condition of a human and of an axolotl. This ambiguity, however, is resolved at the climax and explains the events: *“saber”* (to know) appears to be a similar action among humans or axolotls, with one difference: for the latter, it is a group conscience. That means that intelligibility is a prerequisite and result of the *cogito*; that self-consciousness, considered to be solely attributable to humans, is a shared condition between humans and axolotls:

But that stopped when a foot just grazed my face, when I moved just a little to one side and saw an axolotl next to me who was looking at me, and understood that he knew also, no communication possible, but very clearly. Or I was also in him, or all of us were thinking humanlike, incapable of expression, limited to the golden splendor of our eyes looking at the face of the man pressed against the aquarium. (id., p. 164)

At the end, the axolotl-narrator-self becomes a spectator of the human visitor through a hybrid subjectivity, mainly axolotl but also human. The last shred of doubt about the kind of relationship they have is then dispelled by the materiality of the story itself, written by the man, asked silently by an axolotl to do so.

In a process of gradual rapprochement through sight and eyes, the continuity between species that is initially an anticipated intuition (“because after the first minute I knew that we were linked, that something infinitely lost and distant kept pulling us together.,” id., p. 161) becomes a certainty (“They and I knew.,” id., p. 164). The axolotls-human being’s porosity area is located at the gaze. The verbs see/look/observe already appear in the first paragraph; the amphibians’ eyes seem to be the gateway to a foreign territory (“I tried to see better those diminutive golden points, that entrance to the infinitely slow and remote world of these rosy creatures.,” id., p. 162). Around the verbs “see” and “look” a crescendo is established, which starts with the mere description of the axolotls’ activity (“looking with their eyes of gold at whoever came near them,” id., p. 160), moves on to the spectator’s activity (“lacking any life but looking, letting themselves be penetrated by *my* look, which seemed to travel past the golden level and lose itself in a diaphanous interior mystery,” id., p. 161) and subjectivity (“The eyes of the axolotls spoke to me of the presence of a different life, of another way of seeing,” id., p. 162) and finally reverses the agency terms shortly before the moment of merging (“it was they devouring me slowly with their eyes, in a cannibalism of gold,” id., p. 163). The merging occurs in an eye-to-eye exchange converted suddenly into a dimensional shift:

My face was pressed against the glass of the aquarium, my eyes were attempting once more to penetrate the mystery of those eyes of gold without iris, without pupil. I saw from very close up the face of an axolotl immobile next to the glass. No transition and no surprise, I saw my face against the glass, I saw it on the outside of the tank, I saw it on the other side of the glass. Then my face drew back and I understood. (id., p. 164).

That feeling of terror that Julio Cortázar once declared having felt in the Jardin de Plantes facing the axolotls appears in the story during the brief moment when the narrator mistakenly believes to be trapped in the axolotl’s body, right before he recognizes the continuity between his own subjectivity and that of the other axolotls in the aquarium, as well as between axolotls and man outside the aquarium (cf. id., p. 165). The story does not operate with the usual idea of terror, but with the chilling intuition of a forgotten unity and continuity. The truly disturbing feature of this story is the implementation of the experiment Rosi Braidotti postulates. The merging of human being and axolotls opens up the reader’s imagination to a continuity that confronts the reader with the imposture of human supremacy, thus challenging human uniqueness.

(b) “No se culpe a nadie” (Don’t you blame anyone, 1964)—thing-power and blue wool lust

In one of the most symptomatic studies of new materialism and the material turn in the Humanities, *Vibrant Matter*, the American political theorist and philosopher Jane Bennett meditates on “the curious ability of inanimate things to animate, to act, to produce effects dramatic and subtle” (Bennett 2010, p. 6). In this regard, a very dramatic—even drastic—situation that demostrates the thing-power is refered to in “No se culpe a nadie,” the second tale of *Final de juego*, published for the first time in 1964, in the second extended edition of the book (see above). In it, a blue sweater becomes an actant and a death trap, inducing a man to kill himself by jumping from the twelfth floor of a building.

Bennett includes in her reflections a thinking that goes beyond the living, taking into account the matter that surrounds us and noting how we exchange properties with things (cf. Bennett 2010, p. 10). In this story, Cortázar seems to follow the same impulse, starting from a situation that is well known to all. Who has not once fought a hand-to-hand struggle with a rebellious piece of clothing that refuses to come on, a fight from which we are sure to emerge victorious? Here the expected end is reversed and the melee is won by the usually inert matter, putting its condition into doubt. The object acts in a close, fast and successful fight to death.

In this disturbing takeover of power, the “naturally” inanimate object, a pullover, operates in a situation that is less and less utopian nowadays: the presence not only of inert matter with which we interact in very close contact, to whom we ask questions and expect them to solve problems (computers), but of objects that act on human life, capable of making decisions that may involve the life or death of an individual or groups of human beings. Think of self-driven cars or military robots, such as drones or smart bombs, and the complex ethical debates related to their decision-making.^11^ Thus, the geometry of another reality does not enter into “No se culpe a nadie” through elements that, in 1964, were only imaginable through the literary genre of science fiction,^12^ but through skin contact with the blue wool of a sweater that induces a body transformation and that, finally, provokes the self-destruction of the human being.

As Bennett expresses “thing power may be a good starting point for thinking beyond the life-matter binary, the dominant organizational principle of adult experience” (2010, p. 20). This may also be what the story referred to herein points at: the thing-power opens up a territory of otherness that begins with an ordinary situation. Under “normal” conditions, the blue pullover would be characterized as suffocating; the feeling of drowning in a garment is well-known. But the pullover is given agency and a will of its own. The attribute of suffocating is translated into willingness to suffocate. The pullover’s will is transmitted through contact with the skin, in analogy and yet different from “Axolotl,” where the fusion process starts through visual perception.

The narrative situation is apparently simple. An omniscient extra-diegetic narrator is able to flow together with the protagonist’s subjectivity and read his sensations, thoughts, desires. In its structure, the story is spherical and constituted as a closed parenthesis of action that concludes with the fall of the protagonist into the void. The sphericity is here supported by a striking syntax, which builds up the struggle’s speed. Narrated in a single paragraph marked by rhythm, the story alternates between the protagonist’s difficulties in putting on his pullover and the search for futile rational explanations for what is happening. As the protagonist’s desperation grows, the sentences—very verbal—are prolonged, mimicking and reproducing a feeling of acceleration, logically due to the absence of full stops. In the edition used here, the last sentence, which ends in suicide, takes up 52 lines and is a vortex that leads to the tragic faith. At the end, reference is made to the title, “No se culpe a nadie”/“Don’t you blame anyone,” especially to the word *nadie* (anyone) which points at what should be blamed: the thing.

Jane Bennett wonders if inorganic bodies can also have a life of their own and whether materiality itself can be vital (2010, p. 53). She then argues that ‘objects’ appear as such because their becoming proceeds at a speed or a level below the threshold of human discernment (id., p. 58). So how does Cortázar compose this takeover of a man by a banal everyday object, the level of ‘willingness’ of which is below our perception? The conditions for the awaking of things are equally trivial and already announced in the story’s first sentence: “Cold weather always complicates things a bit, in summer you’re so close to the world, skin against skin […] fall is nothing but putting on and pulling off sweaters, closing oneself in, keeping distances” (Cortázar ([1967] 1998), p. 110). One might say that, in the story, the pullover moves between the human being and the world. Even more: the closer the pullover—which in the course of the story will interweave with the protagonist—the more the world moves away.

From the very first moment, the pullover resists being dressed and immediately produces a passing transmutation of the hand into a claw, which disappears when it is pulled out of the sleeve (“in the evening light the finger looks tinkled and bent inwards, like a black nail with a sharp tip,” id. p. 110). The murderous intent of the pullover can be glimpsed at the very moment the blue wool comes into contact with the skin on the face, nose and mouth (“stifles him more than he could possibly have imagined, forcing him to breathe deeply,” ibid. p. 111). The blue wool acts and interacts with the skin and the protagonist’s sense of taste: “while the wool becomes wet against his mouth, it will probably run and stain his face blue” (ibid.); “there’s also the taste of the sweater, that blue taste of the wool, which is probably staining his face now” (ibid.). The interaction between skin and mouth with the blue wool produces a new tangible reality: “if he opens his eyes his eyelashes bat painfully against the sweater (ibid.).” Skin and wool have interwoven and formed a unit that acts against the protagonist’s will and causes him pain: “[…] the sweater has stuck to his face, with the damp glueishness of his breath mingled to the blueness of the wool, and when the hand pulls upwards it hurts as if something were ripping out his ears and wanting to tear out his eyelashes” (id., p.112). The blue wool takes over the protagonist’s right hand, impeding his liberation from the pullover and finally: “He opens his eyes carefully and sees the five black fingernails hovering over his eyes, and he has just enough time to lower his eyelids and throw himself back” (id., p.113). At the end, the pullover and the protagonist’s own hand have definitely turned against him and the only option is to flee, “to reach at last some place, without the hand, without the sweater” (ibid.). Therefore, the protagonist throws himself out of the 12th-floor window.

From the perspective of new materialism, to work against anthropocentrism means to consider that there is “no longer above or outside of the ‘environment’” (Bennett 2010, p. 120), which, consequently, induces us to contemplate to what extent we are part of a continuity. Although a psychological reading of this story may prevail, the metaphor of the claw, however, arises from the transposition of the qualities of an object into flesh, proposing a fusion of man with the environment, beyond any causality. From this perspective, in “Don’t you blame anyone” Julio Cortázar, a detractor of anthropocentrism, experiments with “a common materiality” and “a wider distribution of agency,” “chasting the fantasies of human mastery,” as Jane Bennett and the new materialists have suggested in recent years (cf. id., p. 122).

## Aesthesis in the almanac books

¿Por qué los analistas literarios tenderán a imaginar en un texto cualquier cosa menos la imaginación?

(Why would literary analysts tend to imagine anything but imagination in a text?, Julio Cortázar, *Último Round,* [1969] 2009a, p. 28)

*La vuelta al día en ochenta mundos* (1967) and *Último round* (1969), the two editorial experiments referred to herein, are part of a series of works in heterogeneous formats, which also includes *Prosa del Observatorio* (1972), *Silvalandia* (1977) and *Territorios* (1978), as well as the book written together with his wife Carol Dunlop *Los autonautas de la cosmopista* (1983). *La vuelta al día en ochenta mundos* and *Último round* are particularly peculiar and symptomatic of Cortázar’s transformative searches; they are characterized by their interesting materiality. The reader is given the same freedom as its author had in making the book: in both works, reading, seeing and perceiving can begin at random and through different images, literary genres and styles. A collage of images and texts overlaps different literary genres in an unusual humorous way. In this mixture of visual language and writing, one encounters things such as poetry, poetic prose, short stories, essays and journalistic articles with different fonts, photographs, nineteenth-century illustrations, drawings, paintings and comics.

Considering the perspective of art history, Luna Chávez differentiates between the formats present in these books and analyzes their relation with artist’s books, object books and the so-called *other* books (cf. 2014, pp. 23–39). The artist’s books appear with the European avant-garde and have great repercussions in Latin America thanks to their dissemination through magazines. In them, artists cross “the borders between visual arts and literature” in order to relate more directly to the recipient (ibid., p. 31). They propose “their own codes for the reorganization of the work” (ibid., p. 32). Dadaism, in particular, offers great space to the absurd and to improvisation, using techniques such as collage and ideograms, as well as new typographies. Surrealism favors the emergence of book-objects, enriching the relationship between poetry and plastic images (cf. ibid., pp. 38–39). With the expression *other books*, Mexican poet and essayist Raúl Renán designated those books that “are not something new, they have always been close to man’s creativity, they prepared the appearance of the formal book” (Renán 1988, p. 19 in Luna Chavez 2014, p. 28). *Other books* are placed outside the usual framework: “works […] that do not meet the conditions required by the industry publishers, but that seek, nevertheless, to leave anonymity” (Renán 1988, p. 14 in Luna Chavez 2014, p. 29). *La vuelta al día en ochenta mundos* (1967) and *Último round* (1969) are situated in the tradition of the avant-garde and their object-books; they are also in line with the artist’s books, as they stand on the border between art and literature; and, finally, because of their genealogy, they are decidedly *other* books. Cortázar refers to them as almanac-books:

Los cronopios y famas, nacidos y escritos en los años cincuenta y comienzos del 60, más textos de un librito que se llama *Un tal Lucas*, también cortos y escritos hace muy poco tiempo, más otros pequeños textos incluidos en lo que llamo libros almanaque (*La vuelta al día en ochenta mundos* y *Último round*), toda esa serie de pequeños textos son mi gran juego personal, mis juegos de niño-adulto-escritor o adulto-escritor-niño. El niño nunca ha muerto en mí y creo que en el fondo no muere en ningún poeta ni en ningún escritor.

(Cronopios and famas, born and written in the fifties and early sixties, plus texts from a little book called *Un tal Lucas*, also short and written very recently, plus other little texts included in what I call almanac books (*La vuelta al día en ochenta mundos* and *Último round*), all that series of little texts are my great personal game, my games as a child–adult writer or adult-writer-child. The child has never died in me and I believe that deep down s/he doesn’t die in any poet or writer., Cortázar in Bernárdez & Álvarez Garriga [1984] 2016, p. 40).

By calling them almanac books, Cortázar places these books within a popular tradition and, thus, assigns them an unintellectual arrangement and a practical use. Like many of his other texts, the almanac books are immersed in a network of intertextual references—and, in these two cases, multimedia references as well—resembling a little compendium of a friends’ universe, Julio Cortázar’s and his artist partner Julio Silva’s, which invites the reader to approach it playfully. Produced in an era of anti-establishment counterculture, they establish an immediate connection with a minor and popular genre; but the term almanac also has an autobiographical relevance and links the books to the annual Peuser Almanac, one of young Cortázar’s favorite readings (cf. Ferro 2018, p. 25; Luna Chávez 2014, pp. 46–47). In an interview given to Saúl Yurkievich and Pierre Lartigue, Cortázar summarized the genealogy and possible categorizations of these texts by stating:

En realidad, esos libros no se proponen como una obra, no tienen la voluntad de totalización propia de una obra. Son una acumulación de texto independientes. Nacen un poco de la nostalgia por esos almanaques de mi infancia, que leían los campesinos y donde hay de todo, desde medicina popular y puericultura hasta las maneras de plantar zanahorias y poemas. La única unidad posible reside en la escritura, proviene de que todos los textos fueron escritos por mí. Estos libros me gustan particularmente porque van contra la noción de género, muy quebrada ya pero que todavía hace estragos. Todavía críticos y lectores se sienten incómodos cuando no pueden clasificar una obra.

(In fact, these books are not proposed as a work, they do not have the will to totalize as a work. They are an accumulation of independent texts. They’re born out of nostalgia for those almanacs of my childhood, which were read by peasants and where there’s everything, from folk medicine and childcare to ways of planting carrots and poems. The only possible unity lies in the writing, which comes from all the texts being written by me. I like these books particularly because they go against the notion of genre, which is already very broken but still rampant. Critics and readers still feel uncomfortable when they cannot classify a work., Cortázar en Yurkievich [1994] 2004, p. 78).

Asked by Arnaldo Orfila Reynal, formerly Fondo de Cultura Económica’s director, if he had any unpublished novel ready after *Rayuela*, Cortázar chose some texts among isolated writings kept in reserve and wrote others specially for the new book, as for instance “relaciones sospechosas” (cf. Letter to Francisco Porrúa of August 22, 1966, Bernárdez and Álvarez Garriga, 2012c, p. 331). Its format was born with the very idea of the book, as Cortázar asked Julio Silva to participate in its layout, “[…] será una especie de almanaque de textos cortos y muy diversos, un libro para cronopios” ([…] which will be a kind of almanac of short and very diverse texts, a book for chronopes., Letter to Julio Silva, August 23, 1966, Bernárdez and Álvarez Garriga, 2012c, p. 333). Through Cortázar’s correspondence, it is possible to get Julio Silva’s answer to his suggestion: “Me emociona y me alegra pensar en trabajar en esto von vos.” (I’m excited and happy to think about working with you., Letter to Julio Silva, September 6, 1966, Bernárdez and Álvarez Garriga, 2012c, p. 334). Silva’s visual proposal is subordinated to the texts, as Silva confirms: “The images always came after [the text], as a support” (Silva in Poll 2013, p. 145). The correspondence between both artists shows, however, their willingness to go through a joint creative process, which would run from the summer of 1966 to December 1967 (cf. Letter to Julio Silva, December 4, 1967, Bernárdez and Álvarez Garriga 2012c, p. 533).

The editorial version of *La vuelta al día en ochenta mundos* that underlies this article, and which coincides in broad terms with the current edition, differs from the first 20 × 24 cm format that delighted both authors. From the beginning, Orfila Reynal and his newly founded publishing house, Siglo XXI, had granted Cortázar great freedoms. A deluxe edition was published in Mexico in 1967, followed by three subsequent editions in Argentina, in 1968, and another three in Mexico in 1969, ending with the seventh and last one, also Mexican, in 1986 (Luna Chávez 2014, p. 52). After December 1970 a popular edition emerges, which, according to Silva, is “una versión amputada” (a cut-down version, Silva in Poll 2013, p. 145), since the graphic components were reduced through the elimination of images, illustrations and ornaments.

*Último round* was also adapted into a simpler version. In its original edition, *Último round* had an elongated format, 16 × 25 cm; the current edition underlying this article is composed of two volumes of 9 × 19 cm. Originally, reading/seeing/perceiving were proposed on two levels: “Primer piso” (first floor) and “Planta Baja” (ground floor). Since 1972, the levels have been erased without separating them into volumes 1 and 2; rather, the two current volumes were rearranged with texts and images from both floors, which means relocating textual and graphic elements and destroying the intended experience, since they are now randomly regrouped by the reader (cf. Luna Chávez 2014, pp. 56–57). This goes for the pocket edition as well.

Although both books have a common format, they differ in terms of their binding theme at composition level (cf. id., pp. 75–156). According to the aesthetic order that organizes the apparent disorder of the almanacs, which is called “rhythm” by Julio Silva (cf. Poll 2013, p. 140), *La vuelta al día* is articulated around the idea of travel, while *Último Round* is a collage. The figure of composition of *La vuelta al día* is, at the same time, the most obvious intertextual reference, which can be read from the title and has great autobiographical weight: it is the work of the other third Julio participating in this voyage, Jules Verne, and his *Vuelta al mundo en ochenta días* (*Le Tour du monde en quatre*-*vingts jours*, 1873). Graphically, Verne’s presence is evident in the nineteenth-century style of the drawings, some directly extracted from his original books. When asked about the very clear reference to Jules Verne in *La vuelta al día*, Julio Silva explains it in terms of Cortázar’s autobiographical context and states that, for the author, imbued since his childhood in a world of books, the French author had the image of a tutor father (Silva in Poll 2013, p. 148).

The process of creating *Último Round* is closely linked to *La vuelta al día*. Initially, it was intended as an additional or second part of the latter; however, in view of its editorial success and of the possibility of publishing the book in foreign languages, Cortázar wrote new texts for it, in an attempt to replace some of the originals with more translatable ones. *Último round* was an unexpected book, since the agreement with Orfila Reynal was limited to *La vuelta al día*. This generated a certain discomfort in the author, perceptible in the explanations that Cortázar provided to his publishing agent and editor in Argentina, Francisco Porrúa (cf. Letter to Francisco Porrúa dated September 23, 1968, Bernárdez and Álvarez Garriga 2012c, p. 624). By that time, however, the title *Último Round* had already been decided upon, after both Julios brainstormed in Cortázar’s summer house at Saignon in Southern France: “Se va a llamar *Último round*, que juega con *round* de box, o sea *vuelta* (última vuelta de la pelea—última vuelta del día, ¿ves?)” (It’s going to be called *Último round*, which plays with a boxing round, that is, a round (last round of the fight—last round of the day, do you see?)., Letter to Julio Silva dated August 28, 1968, Bernárdez and Álvarez Garriga 2012c, p. 611). The most intense writing stage took place during the following Summer in Provence and, by the end of September 1969, the samples were completed (cf. Letter to Julio Silva dated September 25, 1969). Little can be read about the creative process in the correspondence, but it is clear that both authors shared, in Saignon and Paris, stages of deep mutual exchange.

As for *Último Round* being a collage, called by Saúl Yurkievich a literary collage (cf. Yurkievich [1994] 2004: 129–144), a link can be established with *Rayuela*. *Último Round* opens up another level of experimentation through images that not only illustrate the text, but also play with it, question it, complete it, disambiguate it, unite it, fragment it. Below, we perceive not only *Último Round’s* intertextuality, but also its multimedial character, with the collage being defined as part of a Dadaist and Surrealist legacy:

[…] as a symbolic representative of the modern vision, as a matrix image capable of representing that changeable and heteroclite accumulation, that labile interplay of random simultaneities, that chaotic superposition of stimuli that reality has become, a reality that can only be represented through exploded forms and signal heterogeneity. (Yurkievich [1994] 2004, p. 131)

The effects of collage on *Último Round* are even more far-reaching than on *Rayuela*, as this subsequent work introduces not only a mixture of literary genres and visual languages, but also, in its cover and back cover paratexts, news extracts, brief notes, business reviews… all characteristic of gazettes—or of Cortázar’s childhood almanacs. In addition to being an exceptional means of expression “with a playful capacity for action and reaction” (Silva in Poll 2013, p. 142), collage breaks, primarily, with conventional reference frames and encourages seeing/perceiving in a deeper sense. This *other* view of things is part of Cortázar’s avant-garde heritage, which is exemplified by another of his tutelary figures, Jean Cocteau and his work *Opium*, an author’s book that strongly inspired Cortázar (cf. Prego Gadea 1997, p. 68f.). In his article “Estética de lo discontinuo y lo fragmentario: el collage,” (Yurkievich 1984b, pp. 58–73) Saúl Yurkievich relates collage to the art of looking-at-things-differently by stating that:

By dismantling the fixed-point image, by unhinging the chronological and topological successions, by dismembering the extensive harmonic figuration, the collage reorganizes the vision, causes a figurative migration that results in conceptual transmigration, proposes another symbolic scheme to represent the world. (ibid., p. 58)

Thus, although both almanac books are considered marginal works, through their materiality they emerge as paradigmatic examples of the Cortazian universe, which proposes transformation through sensitive experiences, through aesthesis. This idea is present in the books’ artistic, editorial and, of course, literary project.

In recognition of the innumerable associations and exchanges existing between literary and graphic material present in these books, the sequence that extends from the fourth to the fifth chapter of *Last Round*, Volume 1, is henceforth briefly dissected. It includes the diary with poems, called “Uno de tantos días de Saignon” (One of many days of Saignon, Cortázar ([1969] 2009a), pp. 16–39), the poem “Passage d’Oisy” (ibid., pp. 40–41), the short prose “Patio de tarde” (Patio in the afternoon, ibid., p. 42), and the photograph-text “aquí habita la poesía—los cronopios versus el sistema” (here lives poetry—chronopes vs. the system, ibid., p. 43). The sequence is unified at a literary and graphic level by the composition figure “horse.” At the end of the chapter “Uno de tantos días de Saignon,” we see the photograph of a horse (there are signs indicating that it is an Argentinean Creole horse) that looks at the camera; that is to say, it looks at us (ibid., p. 39). The same photograph, yet inverted, illustrates the poem that follows the prose “Passage d’Oisy,” this time in white on a black background. Whoever reads “Patio de tarde,” the next piece of text, will probably become the victim of an illusion, ending up under the impression that the protagonist of the story is a horse. In this brief and ambiguous narrative, one protagonist, Tobi, “remueve la cola” (in Spanish “cola” can mean both glue or tail, “remover” to clear or to stir) as he watches a blonde girl pass through a courtyard. Cortázar’s well-known wink appears only in the last lines, which disambiguate the hypothesis of the horse: “mete el pincel en el tarro y sigue aplicando la cola a la madera terciada” (he puts the brush in the jar and continues to apply glue to the plywood, ibid.). The wink is completed at graphic level at the bottom of page 43’s photograph, which closes the sequence “aquí habita la poesía—los cronopios versus el sistema” (here resides poetry—chronopes vs. the system, ibid., p. 43). The photograph was taken by, among others, a certain “Rocinante” (Don Quixote’s horse) and sent to the narrator in June 1969 by some cronopios from Venezuela (cf. ibid.). The horse/rider composition appears also in the text as a literary motive, announced by the constellation rancho/landscape and, from page 33 onwards, is clearly in correspondence with the couplets (ibid. p. 36 and 37), as well as with the memory of having learned to ride a horse on the back of Prenda (ibid., p. 37).

“Uno de tantos días de Saignon” provides the book with an apparent anchorage in the rural reality of Saignon. It tells the story of its makers: the author and his friends Julio Silva, his English translator Paul Blackburn and Blackburn’s wife wife Joan Miller, all spending a summer day together in Provence. This description includes the postman Monsieur Serre and indirect references to other friends who are far away (Octavio Paz, Christiane Rochefort, Graciela de Sola, Jean Thiercelin, José Lezama Lima, Saúl Yurkievich, Carlos Fuentes). The reader becomes a part of this day, since the prose ends by proposing to her/him: “Y como en algún momento nos iremos a dormir, entonces el poema como almohada para vos que también tendrás sueño, así se acaba este día en Saignon, este grano en una larga espiga de verano” (And since at some point we will go to sleep, then the poem as a pillow for you who will also be sleepy, so ends this day in Saignon, this grain in a long summer ear, ibid., pp. 38–39).

The text is accompanied by photographic sequences, the first suggesting an approach, in three shots, to their house in Saignon (id., p. 19), named by Cortázar “the ranch,” which is the gauchos’ typical building in the Argentine pampas. Another sequence of photos shows the characters of the narration (id., p. 21) and, on the next page, there is a photo of Julio Silva painting a canvas (id., p. 22), followed by a zoom-in picture of his hand in the following page (id., p. 25). On a double page, one can see a photo of a Mediterranean village, probably Saignon (id., pp. 34–35). In spite of its declared anchorage in French Provence, “Uno de tantos días de Saignon” is a cosmopolitan author’s diary about his activities and conversations with intellectual friends of different nationalities and languages. They do not cease to consider current issues, among which Alfred Jarry’s *Père Ubu* and the Bolivian President René Barrientos, which are mixed up with the Vatican’s opposition to the contraceptive pill, the CIA, a soccer game in Buenos Aires, the sunset in Saignon, a tango by Gardel, the verses of José Martí and Atahualpa Yupanqui’s albums, along with numerous literary, artistic and musical references.

The prose has its significant self-reflexive milestone at noon, dispelling the illusion of reality that literary chronology may create: “Será útil indicar que este diario se escribe al final de la jornada y que es un reader’s digest, con perdón de la palabra” (It will be useful to indicate that this diary is written at the end of the day and that it is a reader’s digest, if the pun may be forgiven, id., p. 18). While at a formal level Cortázar tries to permeate the expectations of the reader in terms of literary genres, opening his perception to an array of images, types of letters and diverse literary texts, at the level of content a similar effect is produced here. French Provence appears to be interpenetrated by both the urban cosmopolitan intellectual world and the Argentine pampas of the youthful years of rural teacher Julio Cortázar. Thus, the cosmopolitan inhabitant of a Provence ranch evokes himself as a recent immigrant in France at a café in a corner of Paris 20 years before (in “Passage d’Oisy”), from where he zooms in again, 16 years back, on the pampas, Chivilcoy, the coplas of the gauchos and the Creole horses:

Y si digo al mismo tiempo es cierto, porque en ese París del 51, ya tan lejos del campo argentino del 35, una tarde en que andaba por la rue St.-Ferdinand la sombra de Prenda me cayó encima con su olor a pasto y a cojinillo sudado, entré a un café del lado del Passage d’Oisy y escribí un poema donde todo se daba a la vez, como se me estaba dando…

(And if I say it all at once it’s true, because in that Paris of 51, already so far from Argentine’s countryside of 35, one afternoon when I was walking along rue St.-Ferdinand, the shadow of Prenda fell on me with its smell of grass and sweaty saddle seat cushion, I entered a cafe right by Passage d’Oisy and wrote a poem where everything happened at once, as it came to me., ibid., p. 37)

This is how the “exploded forms and signal heterogeneity” referred to by Yurkievich are reproduced both at a graphic and literary level, in a constant interconnection of fragments that continually creates new meanings. Together, these three texts show an attempt to disarticulate standards, in a fight to broaden the reader’s horizon to new and diverse levels of perception, creating bridges with the public that, in turn, allow them to establish new relationships between its contents.

Hence, what can be said about materiality in Cortázar’s almanac books? What makes them so unique? In their pocket editions—and surely even more in their original first editions—the almanac books can be considered as a coagulated, frozen performance: the book as the result of an experimentation process at a high literary and artistic level. The expression “performance” is used here according to German philosopher Sybille Krämer’s postulate: the idea of the aesthetizing performativity as a dimension of all cultural practices produced from the tension between an event and the perception of it, “to the extent that this relationship can be described in such a way that what an actor produces is received by viewers in a way that goes beyond the symbolism and expressive properties of that performance” (Krämer 2004, p. 21).^13^ This is the case with Julio Cortázar’s and Julio Silva’s multifaceted proposal, which goes hand in hand with a reception that necessarily overflows it. Cortázar would thus describe *La vuelta al día*… to the scholar Graciela de Sola: “Es una especie de baúl, de almanaque. […] En resumen, este libro es un *divertimento*, donde las ilustraciones y el texto juegan un ping-pong que puede agradar al lector sensible” (It is a kind of trunk, a calendar; […] In short, this book is a divertimento, where the illustrations and the text play a ping-pong game that can please a sensitive reader., Letter to Graciela de Sola dated June 3, 1967, Bernárdez and Álvarez Garriga, 2012c, p. 442). The reading is nourished by returning again and again to its poetic, absurd, sensitive, humorous and exciting lines and images, the one and the other in a miscellaneous, fragmented and, yet, intertwined relationship; a ping-pong materiality, exploited and opened up to a sensitive public.

## Footnotes

1 Since Cortázar’s *Último round*, the interviews and his extensive correspondence are only available in Spanish and the present work refers to them, for better understanding, the passages and letter extracts used herein have been translated into English. The author of this article thanks Alexandre Cleaver for his work in reading the manuscript.

2 "Axolotl" is quoted herein according to translation from Spanish by Paul Blackburn, cf. Cortázar [1956] 1998.

3 "Don’t you blame anyone" is quoted herein according to translation from Spanish by Alberto Mangue, lcf. Cortázar [1967] 1998.

4 Cf. Mesa Gancedo (2014, pp. 70–90), here p. 71. As an exception, Sylvie Protin's study “*We band of brothers*: cuerpo y violencia en las traducciones cortazianas de Cocteau y Keats” should be mentioned here, in which the author analyzes Julio Cortázar's translation work through his translation manuscripts (cf. Protin 2019).

5 Joel Vanbroeckhoven reconstructs Cortázar's position back to the Belgian (post-)surrealists, with whom the author clearly agreed more than with the French (cf. Vanbroeckhoven 2019). Based on the aesthetic resources and themes presented in his work, Evelyn Picón Garfield characterized Julio Cortázar in her study as a "Surrealist, in spite of himself" (Picón Garfield 1975, p. 250 in Vanbroeckhoven 2019, p. 41). In this context, it is important to take into account the role of Jean Cocteau, who, as it is well known, never declared himself to be a Surrealist. Jean Cocteau deeply inspired Cortázar through his artistic and human searches (cf. Cortázar’s translations of Cocteau’s oeuvre and his statements about Cocteau’s *Opium*, for instance, in Prego Gadea 1997, p. 69; Bernárdez and Álvarez Garriga 2012a, p. 252; 2012b, p. 607).

6 The II Russell Tribunal was constituted in Brussels on 6 November 1973 and it included, among others, Gabriel García Márquez, Alfred Kastler, Laurent Schwartz and Armando Uribe. The first session of the Russell Tribunal II was held in Rome from March 30 to April 6, 1974, and denounced the violation of human rights in Brazil, Chile, Bolivia, Paraguay and Uruguay. From 11 to 18 January 1975, the second session of the II Russell Tribunal was held in Brussels and it was finally dissolved in Rome at the close of its third session, which occurred from 10 to 17 January 1976.

7 It should be remembered that Jorge Luis Borges appears in the literary vanguard with the ultraist manifesto of May the 20th 1921 that proposed, inter alia, to delete superfluous adjectives in texts.

8 In these days of the coronavirus pandemic is quite shuddering to reread this phrase, isn’t it?

9 Bernardez Albites draws attention to the significant value of axolotls and of the Jardin des Plantes to the West. The latter is the most important botanical garden in France and hosted studies from which a taxonomy of the primitive and exotic was organized. The axolotls exhibited there were the first specimens to be brought to Europe, within the context of the French intervention in Mexico, in 1863. At the time when Cortázar wrote “Axolotl”, the axolotls of the Jardin had been in a state of confinement for almost a 100 years (cf. Bernardez Albites Bernales Albites 2016, pp. 99–100).

10 The first letter in which he refers to the story is dated June 14, 1952. In his previous letter to Jonquières, on 30 May 1952, he makes no reference to his walk in the Jardin.

11 Today, the so-called *Three Laws of Robotics*, formulated by Isaac Asimov in his 1942 short story "Runaround" and which, for decades, served as a guideline for an ethics of the relationship between humans and machines, have been dismissed. Asimov himself had complemented, in *The Robots of Dawn* (1983), his first version by replacing the maxim that forbade a robot of harming a human being with malice aforethought or by inaction with the debatable maxim of not harming humanity, that is, placing the interest of the group above individual interest.

12 From his own statements, it is known that Cortázar was not interested in science fiction, a genre which—with the exception of three or four books he considered indispensable—he declared to be profoundly ignorant of. (cf. Cortázar in Castro-Klarén 1980, p. 15).

13 …und zwar soweit dieses Verhältnis so beschrieben werden kann, dass das, was ein Akteur hervorbringt, von Betrachtern auf eine Weise rezipiert wird, welche die Symbolizität und Ausdruckseigenschafen dieses Vollzugs gerade überschreitet.
